# Acute Lymphoblastic Leukemia Following Nasopharyngeal Carcinoma: Report of an Unusual Case

**Published:** 2017-09

**Authors:** Hamid Farhangi, Mahdi Silanian Toosi, Seied Ali Alamdaran, Sepideh Bagheri

**Affiliations:** 1 *Department of Pediatrics, School of Medicine, Mashhad University of Medical Sciences, Mashhad, Iran.*; 2 *Department of Radiotherapy and Oncology, School of Medicine, Mashhad University of Medical Sciences, Mashhad, Iran.*; 3 *Department of Radiology, School of Medicine, Mashhad University of Medical Sciences, Mashhad, Iran.*

**Keywords:** Acute lymphoblastic leukemia, Children, Chemotherapy, Nasopharyngeal carcinoma, Radiotherapy

## Abstract

**Introduction:**

Nasopharyngeal carcinoma (NPC) is a rare malignancy in children. Nasal obstruction, otitis media with effusion, pain in the ear, hearing problems, and unusual neck mass are among the signs and symptoms of this malignancy.

**Case Report:**

We report the case of a 13-year-old girl with NPC who later developed acute lymphoblastic leukemia (ALL) through the course of her disease. To our knowledge, this is the first report of ALL following childhood nasopharyngeal carcinoma in the English-language literature.

**Conclusion:**

Reports of secondary malignancies at the site of radiotherapy for NPC exist, but this is the first report of ALL following NPC.

## Introduction

Nasopharyngeal carcinoma (NPC) is a rare malignancy in the pediatric population (1), with an annual incidence of 1 per 100,000 children (2). This malignancy is more common in certain parts of the world such as Asia and Africa, probably due to the prevalence of Epstein Barr virus (EBV) infection in these areas (3). 

Pediatric NPCs are most commonly of undifferentiated histology, which is more closely related to EBV infection (1), and affect males and females in a ratio of 2:1 (1). Symptoms are easily confused with cervical lymphadenopathy and unilateral otitis media with effusion (3). 

Intake of preserved foods at an early age, cigarette smoking, and exposure to certain substances such as formaldehyde and wood dust have been linked to increased NPC risk in all populations (4).

Locoregional radiotherapy to the affected area in combination with chemotherapy to reduce distant metastasis is the typical treatment for this malignancy in children (5). Overall survival of children and adolescents with this malignancy has improved over recent decades (6), but the use of intensive radiotherapy in combination with chemotherapy may lead to significant acute and long-term morbidities (5,7).

Here we report a case of pediatric NPC in a child who presented to Dr Sheikh's Children's Hospital in Mashhad, Northeastern Iran. She developed acute lymphoblastic leukemia (ALL) after treatment of NPC.

## Case Report

In July 2009, a 13-year-old girl presented with fever, sore throat, headache, pain in the left ear, and cervical lymphadenopathy. After consideration of her age and clinical presentation, oral antibiotics were initiated. The symptoms, however, persisted and the girl was referred to an otorhinolaryngologist who performed a paranasal sinus computed tomography (CT) scan to rule out infectious complications. 

**Fig 1:**
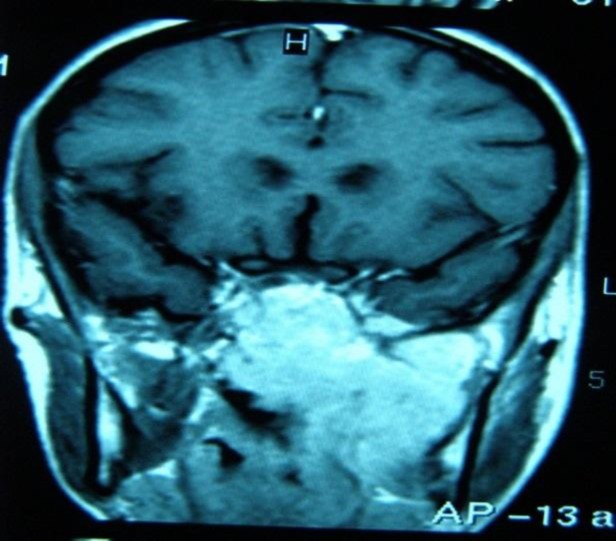
T1 MRI showing a large high-signal soft tissue mass in the nasopharyngeal area and left parapharyngeal space with ill-defined margin and significant airway narrowing

The scan revealed a large, soft tissue mass in the nasopharynx and left paranasal sinus. Magnetic resonance imaging (MRI) was performed to better evaluate the extent of the tumor (Fig.1).

Because histological features of cervical metastases are always identical to the characteristic primary lesions in the nasopharynx, a cervical lymph node excisional biopsy was performed under local anesthesia. This confirmed a high-grade undifferentiated carcinoma. Immunohistochemistry showed a tumor with epithelial-cell origin compatible with nasopharyngeal carcinoma. Considering the tumor size and unilateral cervical lymph node involvement, the girl was diagnosed with stage III nasopharyngeal carcinoma. She had positive serology for EBV, and immunoglobulin M (IgM) anti-viral capsid antigen (VCA) using enzyme-linked immunosorbent assay (ELISA) was positive. A metastatic work-up revealed no evidence of metastasis, except in the left cervical lymph node.

The patient received four courses of chemotherapy consisting of 20 mg/m^2 ^cisplatin for 5 days and 1,000 mg/m^2 ^5-fluorouracil in a 24-hour intravenous infusion for 5 days. This was followed by 68 Gy radiotherapy to the tumor site, 66 Gy radiotherapy to the involved lymph nodes, and 50 Gy to other lymph nodes.

**Fig 2:**
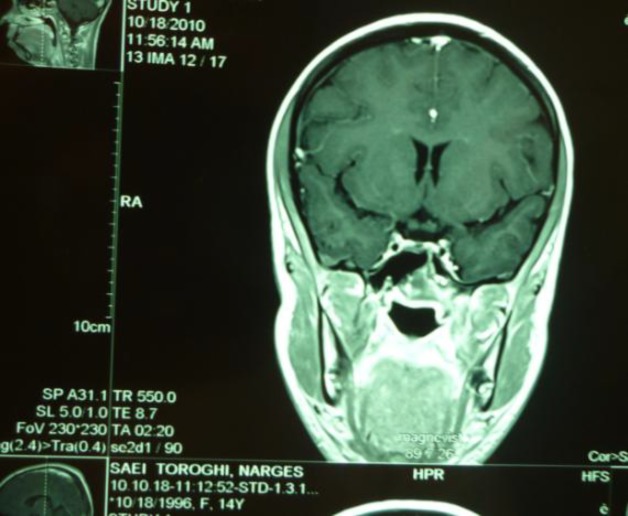
T1 MRI Showing significant resolution of the tumor after radiotherapy.There is only small soft tissue mass in the nasopharynx area, without parapharyngeal involvement

**Fig 3:**
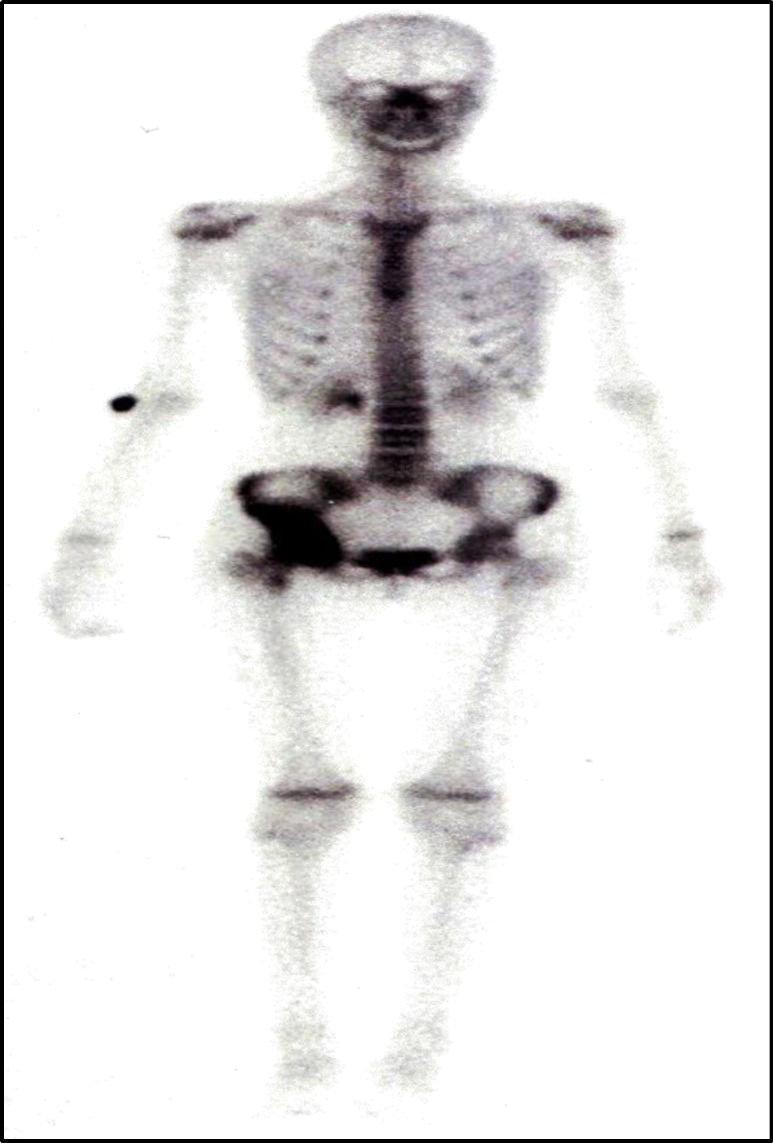
Technetium bone scan showing right pelvic bone increased uptake

MRI after radiotherapy revealed complete resolution of the tumor (Fig. 2.) The patient remained symptom free on follow-up until February 2011 when she presented with a limp and right-side pelvic pain. Plain radiographs of the pelvic bone revealed a right-side lytic lesion. A bone scan was performed (Fig. 3) and a biopsy showed right-sided pelvic bone metastasis of NPC.

The patient received four courses of chemotherapy using the same drugs as previously and 45 Gy radiotherapy to the pelvic metastatic site using external beam radiotherapy with Cobalt 60 unit. Two years later, the patient presented with pallor and bone pain. Complete blood count showed anemia, thrombocytopenia, and leukocytoses with blast cells on a peripheral blood smear. Bone marrow aspiration revealed ALL. Chemotherapy for induction of remission was initiated, but unfortunately the patient died from infectious complications during severe neutropenia.

## Discussion

Childhood and adolescent nasopharyngeal carcinoma is very rare worldwide (1). Because of the similarity of its symptoms with many common childhood infectious conditions, children with NPC are often diagnosed late and tend to have advanced locoregional disease at the time of diagnosis (8,9). Many studies have shown that children and adolescents with NPC respond well to combined modality therapy with chemotherapy and radiotherapy, and this is the most effective method of treatment for childhood NPC (6,8,10-13).

Long-term morbidities have been reported for survivors of childhood NPC, including xerostomia, sensorineural hearing loss, stunted growth, neck fibrosis, thyroid dysfunction, growth hormone deficiency, and secondary malignancies (13-15).

In a study by Liu et al. (9), 27.7% of children with NPC showed treatment failure, with a median failure time of 15 months. The most common site of metastasis was the bone, as in our case. It is noteworthy that our patient’s bone metastasis responded fully to combined treatment with chemotherapy and radiotherapy.

Tumor stage at the time of presentation is one of the most important determinants of survival in NPC patients (9,16,17), highlighting the importance of early detection and diagnosis. A 5-year survival rate of approximately 80% has been reported in NPC patients receiving combined modality therapy (radiotherapy after chemotherapy with cisplatin and 5-FU) (12). Metastases are usually associated with poor prognosis.

 Development of second primary neoplasms in the radiation field has been reported in some studies (6,9), and some studies have reported the presence of a leukemoid reaction in patients with NPC (18-20). However, to our knowledge, development of ALL following NPC has not previously been reported in the English-language literature.

We are not able to explain the etiology in this case, but it could be caused by EBV infection or toxicity related to chemotherapeutic agents, or potentially a genetic predisposition to certain malignancies in this patient.

## Conclusion

In conclusion, most childhood nasopharyngeal carcinomas are already advanced at the time of diagnosis. There are reports of secondary neoplasms at the site of radiotherapy (6,9), but to our knowledge, this is the first report of ALL following NPC in the English-language literature.
